# 青年型肺癌的临床和基因表型特征研究现状

**DOI:** 10.3779/j.issn.1009-3419.2020.101.21

**Published:** 2020-05-20

**Authors:** 萱 韩, 少华 马

**Affiliations:** 100083 北京，北京大学第三医院胸外科 Department of Thoracic Surgery, Peking University Third Hospital, Beijing 100083, China

**Keywords:** 青年型肺癌, 临床特点, 基因背景, 预后分析, Young adult lung cancer, Clinical characteristic, Gene background, Prognostic analysis

## Abstract

青年型肺癌的义为发病年龄≤ 40岁且≥ 18岁的的肺癌患者。与传统肺癌相比，青年型肺癌患者起病隐匿，临床症状不典型，在发现时分期通常较晚，多数出现区域淋巴转移或远处转移。目前研究发现，青年型肺癌具有相对独特的基因背景，其肿瘤驱动基因丰度较高，且与其临床表现和预后有着较为密切的关系。青年型肺癌是近年来肿瘤领域关注的热点，本文就青年型肺癌的临床特征，基因表型特点及预后复习文献并综述，旨在为青年型肺癌的诊疗及临床研究提供一些参考与线索。

## Abstract (en)

Young adult lung cancer is defined as a group of patients refers to whose onset age is less than 40 years old and ≥ 18 years old. Compare with elder lung cancer, the clinical symptoms of them are not typical, the stage is usually late at the time of discovery, and most of them have regional lymphatic metastasis or distant metastasis. Current study found that young adult lung cancer has a relatively unique genetic background, the abundance of tumor-driving genes is high, and it is closely related to its clinical manifestation and prognosis. Young adult lung cancer is the focus of attention in the field of cancer in recent years. This article reviewed the literature on the clinical features, gene phenotypic characteristics and prognosis of young adult lung cancer in order to provide provide some references and clues for the study on young adult lung cancer.

肺癌全球肿瘤发病率位居第二，死亡率均位居第一，在我国两者均为首位，平均发病年龄为72岁^[1]^。而在临床工作中发现，肺癌中年轻患者比例有增加趋势。检索文献表明，绝大多数研究将年龄≤ 40岁且≥ 18岁的肺癌患者定义为青年型肺癌。青年型肺癌发病很多是局部晚期或者远处转移^[2]^，但与老年型肺癌预后比较又存在争议^[3, 4]^。青年型肺癌基因突变率及突变丰度均较高，且以间变性淋巴瘤激酶(anaplastic lymphoma kinase, *ALK*)重排最常见^[5, 6]^。由此可见，青年型肺癌可能是一组较为特殊的病例。本文就青年型肺癌的临床特征、预后情况、及基因为背景做一综述。

## 1 青年型肺癌的临床特点

肺癌是一组异质性疾病，尤其是非小细胞肺癌(non-small cell lung cancer, NSCLC)，其临床特征多变，多数患者症状不典型。青年型肺癌也同样具有起病较为隐匿，症状表现不规律，具有容易被忽视和误诊的共性。但青年型肺癌也有其独特的表现。

目前大多数文献指出，青年型肺癌总体发病率占所有肺癌患者的2%^[2, 7, 8]^，女性发病率高于男性，在女性患者中，无吸烟史者所占比例较高^[2]^。与年龄 > 70岁肺癌比较，体力状况(performance status, PS) =0分或1分，合并症较少，尤其是呼吸系统合并症。青年型肺癌在不同人种中的分布也不相同，但相关报道较少。Ramalingam等^[9]^在1998年发表的一篇基于监测、流行病学及预后(Surveillance, Epidemiology, and End Results, SEER database)对386例青年肺癌患者的回顾对照研究中指出，在美国、非裔黑人中青年型肺癌患者较其他人群更为多见，并认为这与社会因素相关，如非裔人群的居住环境较差、易受环境危险因素暴露、及非裔人群的吸烟比例较高等。Sheyhidin^[10]^在一项针对我国新疆不同民族青年型肺癌的分析也指出，不同民族间发病情况不同，可能与不同民族间饮食、居住环境、及文化背景相关。除去社会学因素，人种基因背景与种族间发病差异率是否存在联系尚未有明确结论，需要更多的研究证实。

青年型肺癌患者发病时临床分期通常较晚，大多初诊时即为Ⅲ期-Ⅳ期，表现为肿瘤细胞区域淋巴结转移活跃，以及更多的远处转移。光镜下，肿瘤病理多数为低分化腺癌。Xia等^[10]^利用SEER的肺癌研究发现：与老年肺癌患者相比，青年型肺癌患者的淋巴结转移率更高；无论T分期如何，区域淋巴结阳性率随着年龄的增加而降低；同时该研究认为发病年龄小可以作为评估肺癌淋巴转移的独立预测因子，提示了青年型肺癌患者术前应当充分评估区域淋巴结情况。青年型肺癌的远处转移也更为明显，其中，脑转移为最常见的远处转移。Suidan等^[8]^的研究表明出现脑转移的青年肺癌患者显著多于老年患者(39% *vs* 25%, *P* =0.04)，但两者在颅内转移瘤的位置、转移灶的数目方面无明显差异。

有趣的是，如同肺癌本身是一种异质性疾病一样，不同年龄的青年型肺癌患者的临床特征也并非完全一致，Liu等^[12]^的研究表明：通过将4, 623例青年NSCLC患者分为年幼组(18岁-30岁，*n* =429)和年长组(31岁-40岁，*n* =4, 194)，对比两组肿瘤组织病理发现：年幼组中高分化肿瘤的比例相对较高，而年长组中低分化肿瘤所占比例相对较高；两组间肿瘤分期也有所差异，年幼组患者中Ⅰ期患者较多，而年长组中Ⅲ期患者较多，两组中Ⅱ期、Ⅳ期比例相当。这与发病年龄与肿瘤细胞成熟性呈正相关的观点不同，该研究暗示青年型肺癌本身也不应当视为一种同质性的疾病。

既往研究认为青年型肺癌患者临床分期较晚的原因一方面是青年患者起病较为隐匿，症状不典型，容易误诊，另一方面是由于吸烟史和环境毒素暴露所致。但最新的调查研究表明，青年型肺癌女性患者中吸烟比例反而较低^[2]^，且随着低剂量计算机断层扫描(computed tomography, CT)的普及应用，更多的肺癌被早期发现。因此，无法完美的解释青年型肺癌以晚期多见的临床现象。随着对基因背景和肿瘤行为间联系的深入认识，越来越多的研究认为青年型肺癌分期较晚可能与其特殊的基因背景相关，其肿瘤驱动基因丰度可能更高，肿瘤侵袭行为可能更为活跃，而导致初始发病即表现为局部晚期或出现远处转移。

## 2 青年型肺癌的基因背景差异

青年型肺癌的早期文献很少对肿瘤的基因背景进行探究，一方面是因为受限于当时对肺癌驱动基因的认识，另一方面是早期的数据库资料缺乏对相关基因的统计。随着对肺癌基因背景研究的不断深入，2010年后较多的青年型肺癌的研究开始着眼于对肿瘤驱动基因的探究。

表皮生长因子受体(epidermal growth factor receptor, *EGFR*)突变和*ALK*重排是经典的肺癌肿瘤驱动基因，探究两者在青年NSCLC患者和老年NSCLC患者肿瘤组织间基因丰度是否存在差异，可以反映基因背景在青年型肺癌中的所扮演的角色。日本学者Nagashima等^[15]^对12例青年型肺癌患者进行基因检查发现，12例患者中4例存在*EGFR*突变，7例EGFR阴性患者中，5例存在*ALK*重排。Adrian等^[16]^发表在*JAMA Oncol*上的文章旨在探究NSCLC患者年龄和靶基因突变间的关系，通过对2, 237例肺癌患者(其中青年型肺癌患者81例)进行回顾对照研究，研究发现50岁以下的肺癌患者与老年患者相比基因改变的发生率增加了59%，提出从基因突变背景上可以将50岁作为青年型肺癌的年龄切点，这与传统上将青年型肺癌定义为40岁以下患者不同。在众多高突变率的基因中，*ALK*重排与青年型肺癌发病关系最为密切。此外，台湾学者Hsu等^[17]^源自SEER数据库的研究指出青年型肺癌患者的*EGFR*突变率明显低于老年患者(52.5% *vs* 60.6%, *P* =0.001)，而在野生型EGFR患者中，同样发现青年型肺癌患者的肿瘤组织检测出更高的*ALK*重排率。浙江大学的Yang^[6]^团队对640例肺癌肿瘤组织(其中青年型肺癌54例)进行*EGFR*、*KRAS*、*NRAS*等突变基因检测和*ALK*、*ROS1*及*RET*等融合基因检测发现，不同年龄组间*ALK*、*ROS1*及*RET*等融合基因的发生率差异较为显著(22.2% *vs* 4.1%, *P* < 0.001)，而*EGFR*、*KRAS*、*NRAS*等突变基因的发生率则无明显差异(46.3% *vs* 45.6%, *P* =0.92)。日本学者Tanaka等^[5]^的研究发现青年型肺癌的ALK阳性率较老年型肺癌明显增高(41% *vs* 4%, *P* < 00.001)。这些结果从一定程度侧面反映了相较于*EGFR*突变，*ALK*基因重排在青年型肺癌患者肿瘤的基因背景中扮演了更重要的角色。

考虑到*ALK*基因重排在全部肺癌驱动基因中所占比例并不高，故有必要探究青年型肺癌中*ALK*重排率与NSCLC患者整体的*ALK*重排率相比差异是否显著。Corrales-Rodriguez^[18]^利用拉丁美洲肺癌调查中心的数据，对拉丁美洲青年型肺癌的流行病学研究中，青年型肺癌患者的肿瘤组织中ALK重排率为10.1%，拉丁美洲肺癌患者中*ALK*重排率为6.55%，在既往文献中，不分种族和人群的*ALK*重排发生率为2%-13%。这也反映了青年型肺癌可能与较高的*ALK*重排率相关。其他国家和地区中，尤其是*ALK*基因阳性率相对较低的东亚地区，青年型肺癌患者的*ALK*基因融合易位是否显著高于普通肺癌人群，则需要更多的研究证实。

目前一些研究对青年型肺癌的基因背景提出了不同的观点。2019年，Yang等^[7]^对20例青年型肺癌患者的肿瘤组织进行体细胞突变检测，通过联合分析24例对照组老年患者的肿瘤组织及正常样本序列，从而检测出青年型肺癌患者肿瘤细胞DNA上发生的点突变和插入/缺失突变。但结果表明两组间体细胞突变检测无显著差异。此外，该实验中，两组间*ALK*重排和*EGFR*突变、*K-RAS*突变的发生率无显著差异。与发病年龄较小相关性联系最为紧密的两个基因是*FRG1*和*KMT2C*。文章同时指出了在不吸烟的青年型肺癌患者中检测出了相对较低的*KMT2E*基因突变。此外，与普遍东亚人种肺癌的基因背景相比，青年型肺癌在*TP53*、*TGFBR2*、*MLH3*和*ELAC2*上有较高突变率。

越来越多的研究着眼于对青年型肺癌基因背景的探究，但目前对肺癌基因背景的研究依旧是冰山一角，而青年型肺癌是否存在特异的驱动基因仍然需要进一步的研究证实。

## 3 青年型肺癌的远期生存状况

目前肺癌的5年生存率约9%-20%^[1]^，即使手术技术、新辅助化疗、靶向药物治疗多学科策略日新月异，肺癌的预后仍不容乐观。

与老年患者相比，青年型肺癌在肿瘤行为上更具有侵袭性，临床分期更晚，其预后理应较差^[3, 19]^。然而近来Yoneyama等^[2]^对266例青年型肺癌患者进行生存分析表明，青年型肺癌患者的1年、3年、5年总体生存率分别为82.9%、64.6%和57.0%，显著高于对照组的老年肺癌患者。这可能是由于青年型肺癌有独特的基因背景、PS评分较低、更少的合并症、更积极的术后化疗及靶向治疗等综合因素相关，继而表现出了较好的预后。

对青年型肺癌预后的研究是一个曲折的过程。2010年之前的研究普遍认为青年型肺癌患者即使有着较低的PS评分，合并症更少，且接受更加积极的以放/化疗为主的围术期综合治疗，相较与老年肺癌患者并未表现出更好的预后，甚至更差。这与青年型肺癌患者起病隐匿，临床分期通常较晚且对放化疗不敏感有关。2001年，日本学者Maruyama等^[14]^指出接受手术的青年型肺癌患者的总体生存率与老年肺癌患者无明显差异，对于青年肺癌患者的治疗仍应当遵循美国国立综合癌症网络(National Comprehensive Cancer Network, NCCN)指南进行。2004年，复旦大学的Chen等^[14]^团队的对照研究表明，在基于铂类化疗和放疗对NSCLC患者的治疗中，青年型肺癌患者与其对照组相比，即便予以更加积极的放/化疗，也并未表现出更好的总体生存率。2006年，在希腊学者Mauri等^[21]^的研究中，其中青年型肺癌患者的中位生存时间为12个月，而对照组中老年肺癌患者为11.5个月。两组间总体生存率无显著差异。在接受一期化疗患者中，青年型肺癌患者的疾病进展时间(time to progress, TTP)为4.3个月(95%CI: 3.2-5.3)，对照组的TTP为5.8个月。虽然两组差异具有显著性，但两组的TTP均较短，预后不佳，无法说明问题。在接受姑息性化疗的患者中，青年型肺癌患者的TTP为2.7个月(95%CI: 0-6.5)，对照组TTP为4.4个月(95%CI: 3.2-5.5)，两组间无显著差异。

2010年以来，随着临床研究对肺癌驱动基因认识的不断深入，越来越多的肺癌靶向药物，如酪氨酸激酶抑制剂(tyrosine kinase inhibitor, TKI)、ALK抑制剂如克唑替尼等应用于临床治疗当中。更多的临床研究证实，青年型肺癌患者的预后优于老年患者。2015年，Rich等^[22]^利用英国国家肺癌数据库(National Lung Cancer Audit dataset, NLCA)对青年型NSCLC患者的研究中发现，与老年肺癌患者相比，青年型肺癌患者接受手术的比例高出66%，其围术期死亡率仅为对照组老年患者的一半，此外，青年型肺癌患者对术后综合治疗的反应也相对较好。同年，Thomas等^[23]^的研究在肺癌特异性生存(lung cancer specific survival, LCSS)方面着眼，结果表明了青年型肺癌的患者的OS及LCSS均好于老年患者，包括出现远处转移的患者。

一些临床研究证实了青年型肺癌患者对靶向药物更为敏感，表现出了更好的生存和预后，这与青年型肺癌患者肿瘤组织中肿瘤驱动基因丰度更高是密切相关的。2017年，Tanaka等^[24]^的研究通过检测肺癌患者肿瘤组织中驱动基因突变的频率，发现驱动基因突变频率较低的青年型肺癌患者，其中位生存时间明显短于对照组中的老年患者(8.9个月*vs* 16.4个月，*P* < 00.01)。此外，驱动基因突变频率高的青年型肺癌患者的中位生存时间为24.9个月，与其他对照组相比，在总生存期和预后方面表现出了明显优势。2018年，Pan等^[25]^对252例青年型NSCLC患者的生存分析中发现，在接受化疗的患者中，无进展生存期(progression-free survival, PFS)和OS分别为3.3个月和27.6个月，而接受EGFR-TKIs治疗的患者，其PFS和OS分别为12.1个月和33.6个月，均显著延长。在*ALK*阳性的青年肺癌患者中，早期接受克唑替尼者，PFS为21.9个月，预后得到明显改善。

年轻型肺癌的预后相对较好，靶向治疗能够为其预后带来更高的收益，但不可否认的是，对于Ⅳ期肺癌患者，其预后结局依然不容乐观。青年型肺癌治疗的个体化研究需要进一步探索。此外，目前尚无研究对青年肺癌靶向药物的耐药性进行研究。青年型肺癌患者独特的基因背景是一把双刃剑，肿瘤组织中丰度较高的驱动基因和较高频率的基因突变，是否会增加靶向药物的耐药几率，对青年型肺癌患者的预后造成影响，也需要进一步的研究和证实。

## 4 目前青年型肺癌研究的局限性

目前青年型肺癌的临床研究仍处于探索阶段，虽然现阶段的文章和研究均支持青年型肺癌具有独特基因背景及*ALK*阳性在青年型肺癌驱动基因中扮演了重要角色，但受限于当前临床研究条件和对青年型肺癌驱动基因致病机理的认识不足，目前研究仍具有一定的局限性。第一，由于青年型肺癌在全部肺癌中所占比例仅2%，且发病隐匿，在临床上并不多见，导致目前的临床单中心研究所纳入的样本量较少，不同样本来源的研究可能得出不同的结论。第二，在临床治疗中，患者年龄、经济条件等社会因素也影响了肺癌患者的预后。第三，青年型肺癌的研究受限于目前对肺癌驱动基因的认识，经典的肺癌驱动基因如ALK阳性在青年型肺癌疾病进程中是否存在决定性作用目前尚无定论，而是否存在其他更独特的致病基因，仍然需要今后的大宗试验和研究证明。
